# Prospects for combining immune checkpoint blockade with PARP inhibition

**DOI:** 10.1186/s13045-019-0784-8

**Published:** 2019-09-14

**Authors:** Anping Li, Ming Yi, Shuang Qin, Qian Chu, Suxia Luo, Kongming Wu

**Affiliations:** 1 Department of Medical Oncology, The Affiliated Cancer Hospital of Zhengzhou University & Henan Cancer Hospital, Zhengzhou, 450008 China; 2 Department of Oncology, Tongji Hospital of Tongji Medical College, Huazhong University of Science and Technology, Wuhan, 430030 China

**Keywords:** PARP inhibitor, DNA damage response, PD-1, PD-L1, CTLA-4, Immunotherapy, Combination therapy, Tumor immune microenvironment

## Abstract

The immunogenicity of a cancer cell is derived from accumulated somatic mutations. However, on the contrary to increased immunogenicity, anti-cancer immune response tends to be feeble. This impaired anti-cancer immunity could be attributed to multiple factors including loss of immunodominant epitopes, downregulation of major histocompatibility complex, and immunosuppressive microenvironment, as well as aberrant negative co-stimulatory signals. Immune checkpoint inhibitors block negative co-stimulatory signals such as programmed cell death-1 and cytotoxic T-lymphocyte-associated protein 4, ultimately reactivating anti-cancer immunity. Immune checkpoint inhibitors elicit potent anti-cancer effect and have been approved for multiple cancers. Nevertheless, there still are significant potential improvements for the applications of checkpoint inhibitor, especially considering frequent resistance. Recent studies demonstrated that additional PARP inhibition could alleviate resistance and enhance efficacy of immune checkpoint blockade therapy via promoting cross-presentation and modifying immune microenvironment. We proposed that PARP inhibitors could enhance the priming and tumor-killing activities of T cell, boost the whole cancer-immunity cycle, and thereby improve the response to immune checkpoint blockade. In this review, we focused the latest understanding of the effect of PARP inhibitors on anti-cancer immunity and PARP inhibitors combining immune checkpoint blockade therapy. Moreover, we summarized the preclinical and clinical evidence and discussed the feasibility of this combination therapy in future clinical practice.

## Background

Cancer cells harbor substantial gene mutations and possess abnormal protein expression pattern. According to the specificity of expression, aberrantly generated proteins could be classified as tumor-associated antigens (TAAs) and tumor-specific antigens (also called neoantigens) [1–3]. TAAs refer to proteins remarkably overexpressed on cancer cells compared with normal cells. Neoantigens are proteins exclusively expressed on cancer cells due to mutation-mediated sequence alterations [4, 5]. TAAs and neoantigens determine cancer immunogenicity and initiate the cancer-immunity cycle [6]. Although host immunity could theoretically recognize tumor-derived materials and retard tumor growth, a subset of cancer cells escape from immune surveillance and develop into visible tumor lesions [7].

An effective anti-cancer immune response relies on the robust cascade reaction including release and presentation of cancer antigens, priming and activation of T cells, trafficking and infiltration of T cells, and recognizing and killing tumor cells [8]. However, one or more steps of this cancer-immunity cascade reaction are undermined in cancer patients. A growing body of evidence indicated that loss of cancer-specific immunodominant epitopes and T cell repertoire, downregulation of antigen processing, and presentation machinery, as well as immunosuppressive microenvironment could lead to immune tolerance to tumor antigens [9, 10]. Immunotherapy restores or enhances anti-cancer immune response via eliminating inhibitory immune components, transferring additional tumor-specific T cell clones, reshaping immunosupportive microenvironment [11–14]. Immune checkpoint inhibitor (ICI), cancer vaccine, and adoptive T cell transfer have been applied in multiple cancers [15–18]. Nevertheless, due to the spatial heterogeneity and dynamically evolving cancer antigen spectrum, it is hard to cure a tumor by monotherapy and tumors cells eventually acquire resistance [19]. Therefore, immunotherapy-based combination strategy attracts extensive attention for synergistic efficacy and lower risk of resistance [20, 21].

Poly ADP-ribose polymerase (PARP) inhibition induces synthetic lethal effect in cancer cells with a deficiency in homologous recombination (HR) [22]. Besides, PARP inhibitor (PARPi) could promote the priming of anti-cancer immune response and enhance Th1-skewing immunity, as well as modulate immune microenvironment [23]. The immunological effect of PARPi is multifaceted which might be favorable to boost cancer-immunity cycle and enhance the efficacy of ICI treatment [24]. This review focused on preclinical studies and clinical trials of PARPi combined with ICI therapy, as well as prospects and challenges of this combination therapy.

## PARP inhibition

### The role of PARP in DNA damage response

Genome intensity is challenged by continuous DNA damage events [25]. Normal cells could detect and repair DNA damages by multiple pathways: (1) DNA single-strand break (SSB) repair pathways including base excision repair (BER), nucleotide excision repair (NER), and mismatch repair (MMR) and (2) DNA double-strand break (DSB) repair pathways such as HR and nonhomologous end joining (NHEJ) [26]. For cells harboring mutations in gene coding, the key components of DNA damage response (DDR) such as BRCA1/2, TP53, and MSH2, inadequate elimination of genome mutations increases the risk of carcinogenesis after DNA damage events [27]. Actually, cancer cells often possess inadequate repertoire of DNA damage repair pathways and highly depend on certain DNA repair pathways to avoid lethal DNA damages [27, 28].

**Fig. 1:**
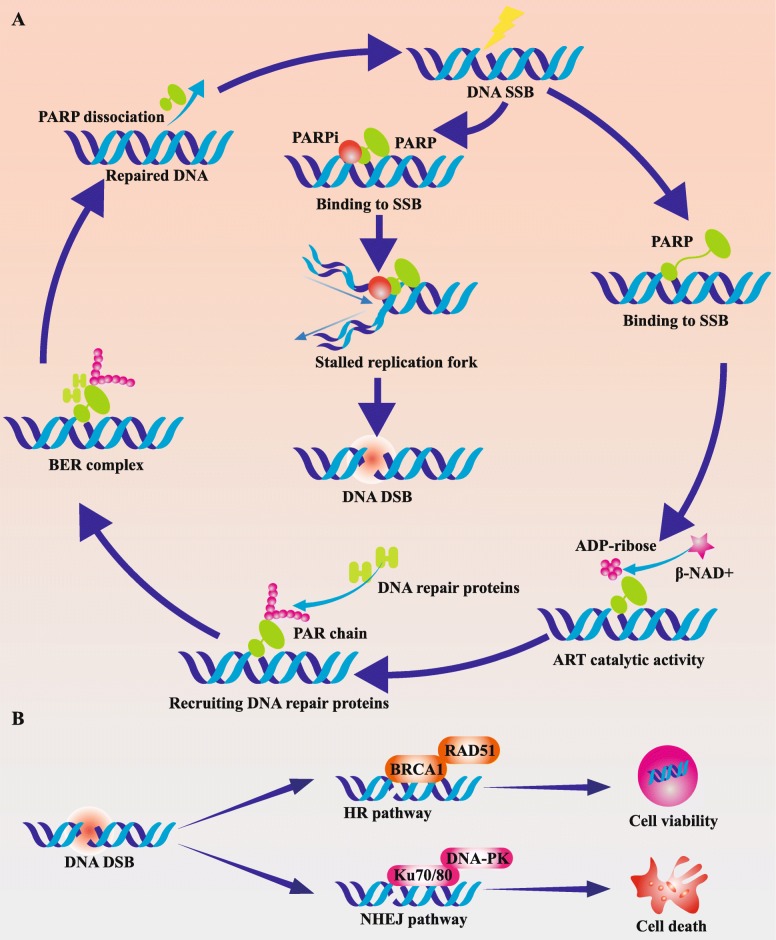
DNA single-strand break and double-strand break repair pathways. **a** PARP catalytic cycle and PARP inhibitor. PARP is a core DNA damage sensor in DDR, which binds to damaged DNA lesions, catalyzes the generation of negatively charged poly (ADP-ribose) chains, remodels the structures of damaged chromatin, and recruits DNA repair-related protein complex. Then, PARP is dissociated from DNA damage site by auto-PARylation. PARPi could interfere with the interaction between PARP and its cofactor (β-NAD), inhibit PARylation activity, and trap PARP on damaged DNA chain. **b** Double-strand break repair pathways. In normal cells, when both HR and NHEJ pathways are available in G2/M stage, HR pathway is preferentially adopted to repair DSB. HR is an effective repair approach with high fidelity which uses the sister copy of damaged sites as the template. However, for some cancer cells with HR deficiency such as BRCA1/2 mutations, NHEJ pathway is utilized for DSB repair. NHEJ is an error-prone repair pathway with low-fidelity which could induce unsustainable DNA damages (e.g., chromosomal rearrangements and mutations) and eventual cell death. β-NAD, β-nicotinamide adenine dinucleotide; DDR, DNA damage response; NHEJ, nonhomologous end joining; HR, homologous recombination; SSB, single-strand break; DSB, double-strand break

PARP is a core DNA damage sensor in DDR, which binds to damaged DNA lesions, catalyzes the generation of negatively charged poly (ADP-ribose) chains, remodels the structures of damaged chromatin, and recruits DNA repair-related protein complex [29, 30]. Then, PARP is dissociated from the DNA damage site by auto-PARylation [31]. It has been well established that PARP mainly participates in BER-mediated SSB repair, as well as other multiple DDR pathways (Fig. 1a) [32].

### PARPi and synthetic lethal effect

The anti-cancer effect of PARPi has not been completely understood yet, which is initially attributed to inhibition of catalytic effect [33]. As a result, SSB persists and eventually develops into replication-dependent DSB [34]. In normal cells, when both HR and NHEJ pathways are available in G2/M stage, HR pathway is preferentially adopted to repair DSB [35]. HR is an effective repair approach with high fidelity which uses the sister copy of damaged sites as the template [36]. However, for some cancer cells with HR deficiency such as BRCA1/2 mutations, NHEJ pathway is utilized for DSB repair [37]. NHEJ is an error-prone repair pathway with low fidelity which could induce unsustainable DNA damages (e.g., chromosomal rearrangements) and eventual cell death (Fig. 1b) [38]. Based on this synthetic lethal effect, numerous PARPis are developed including Veliparib, Rucaparib, Olaparib, Niraparib, and Talazoparib, which are mainly applied in cancer patients with BRCA1/2 mutations [39–44].

Then in clinical practices, some phenomena emerged which could not be fully explained by synthetic lethal theory. Firstly, the capability of PARPi to inhibit PARP catalytic activity is not closely correlated to its cell-killing ability in BRCA-mutated tumors [32]. Besides, PARPi could induce greater tumor-killing effect than PARP depletion [32]. Actually, these phenomena are attributed to the PARP trapping potency of PARPi. In general, all agents belonging to PARPi could interfere with the interaction between PARP and its cofactor (β-nicotinamide adenine dinucleotide), inhibit PARylation activity, and trap PARP on damaged DNA chain [45]. Apart from uncontrolled DNA damage accumulation, DNA-PARP complex-mediated cytotoxicity also contributed to tumor cell death [46]. Originally, the potent anti-cancer effect of PARPi was found in BRCA1/2 deficient ovarian cancer patients. Later, the clinical use was expanded to BRCA-mutated breast cancer, ovarian cancer, pancreatic cancer, prostate cancer [47–49]. Recently, it was found that some non-BRCA-mutated tumors shared therapeutic vulnerabilities with BRCA-mutated tumors [50]. These non-BRCA-mutated tumors termed as BRCAness tumors that often harbor other alterations in HR genes except for germline BRCA deleterious mutations [50]. Patients with BRCAness tumors could benefit from PARPi treatment as well [50].

In spite of the great success of PARPi in a particular population, there are some problems needing to be properly resolved, especially acquired PARPi resistance [51]. This resistance is primarily attributed to the inactivation of key molecules involved in NHEJ pathway, such as 53BP1, the loss of PARP, and secondary mutations in BRCA restoring the activity of HR [52–54]. Combination therapy is a feasible strategy to enhance efficacy and decrease treatment resistance. It is notable that PARPi combining chemotherapy is easy to induce dose-limiting toxicity [55]. In the meanwhile, based on the hypothesis that patients with mutations in BRCA1/2 or other HR components are prone to possess higher mutation burden, multiple clinical trials are ongoing to explore the efficacy of combination therapy of PARPi and immune checkpoint inhibitors [56]. The interim analysis of SOLO1 (NCT01844986) showed that BRCA1/2-mutated ovarian cancer patients could significantly benefit from Olaparib treatment (hazard ratio of cancer progression or patient death = 0.30, 95%CI 0.23–0.41, *P* < 0.001) [57]. In another phase III study POLO (NCT02184195), 154 germline BRCA1/2 mutant pancreatic cancer patients were enrolled [58]. The results of the POLO study indicated that patients undergoing Olaparib treatment tended to have a prolonged progression-free survival than the placebo group (median progression-free time: 7.4 months vs. 3.8 months, hazard ratio = 0.53, 95% CI 0.35–0.82, *P* = 0.004) [58].

## Immune checkpoint inhibitor

Two signals are required for the activation of naïve T cells [59]. The first signal is the specific binding between T cell receptor (TCR) and antigenic peptide-major histocompatibility complex (pMHC) [59]. Contrary to the first signal, the second signal is a non-antigen-specific pathway which depends on the binding between co-stimulatory molecules and corresponding ligands [60]. The balance between positive and negative co-stimulatory signals is crucial for the activation and tolerance of T cells [61, 62]. Among negative co-stimulatory molecules, programmed cell death-1 (PD-1) and cytotoxic T-lymphocyte-associated protein 4 (CTLA-4) are relatively well-studied signals which are also termed as immune checkpoints.

### PD-1/PD-L1 signaling pathway

PD-1 is expressed on multiple activated immune cells including T cells, B cells, natural killer (NK) cells, and dendritic cells (DCs) [63]. As the main ligand of PD-1, PD-L1 is constitutively expressed on a wide variety of immune cells and non-immune cells [63, 64]. Besides, the expression of PD-L1 could be induced by inflammation response [65]. In the context of TCR stimulation, the immunoreceptor tyrosine-based inhibitory motif (ITIM) and immunoreceptor tyrosine-based switch motif (ITSM) of PD-1 are phosphorylated which further recruit SHP1/2 and counteract TCR/CD3-CD28 mediated tyrosine phosphorylation [66, 67]. As a result, the downstream signaling cascade of TCR is inhibited by PD-1/PD-L1 axis. Besides, PD-1/PD-L1 signal could also suppress intercellular PI3K-Akt and Ras-Raf-MAPK signaling pathways, which further downregulates glycolysis, the metabolism of amino acid and fatty acid oxidation, as well as cell proliferation [68, 69]. Dysregulated metabolism promotes the differentiation of T cells towards regulatory T cells (Tregs) [70].

### B7-CTLA-4 signaling pathway

CTLA-4 is a negative co-stimulatory molecule which mainly regulates the priming and activation of T cells in peripheral lymphatic organs [6]. CTLA-4 is constitutively expressed on Tregs and transiently upregulated on conventional T cells after activation [71]. CTLA-4 competitively antagonizes CD28 by binding to CD80 (B7.1) and CD86 (B7.2) [72]. Subsequent internalization of CD80/CD86-CTLA-4 complex decreases the abundance of available co-stimulatory molecule ligands and elevates the threshold of T cell activation [73]. Besides, through intracellular ITIM, CTLA-4 could counteract TCR/CD3-mediated tyrosine phosphorylation and inhibit the signal transduction of TCR [74].

### Clinical application of ICI

Since the first ICI was approved for metastatic melanoma patients in 2011, numerous ICIs have entered clinical practice [75, 76]. Anti-CTLA-4 and anti-PD-1/PD-L1 treatment exhibited a potent and durable tumor-killing effect in multiple advanced cancers such as triple-negative breast cancer, non-small lung cancer, renal cell cancer, melanoma, and urothelial bladder cancer [77–81]. However, the clinical application of ICIs is limited by low response rate [82]. Although a series of biomarkers have been adopted to predict the efficacy of ICI and select patients before treatment beginning, the actual primary and acquired drug resistance has not been completely overcome [17]. Some factors have been verified as the core determinants of the efficacy of ICIs treatment such as tumor mutation burden, MMR deficiency, the status of tumor-infiltrating lymphocytes (TILs), PD-L1 expression, and immunosuppressive microenvironment [83–86].

The development of ICI-based combination therapy provides a novel perspective to enhance ICI efficacy and overcome treatment resistance. ICIs are usually combined with chemotherapy, radiotherapy, and targeted therapy, as well as antiangiogenic therapy [20, 87–89]. Generally, the combination therapy is aiming to promote antigen presentation, broadening T cell repertoire, and impairing immunosuppressive components [90]. The results of NCT02763579 showed that atezolizumab plus cytotoxic chemotherapy (carboplatin and etoposide) showed more potent anti-cancer effect than chemotherapy in advanced small cell lung cancer patients (median overall survival 12.3 months vs. 10.3 months, hazard ratio = 0.70, 95% CI 0.54–0.91, *P* = 0.007; median progression-free survival 5.2 months vs. 4.3 months, hazard ratio = 0.77, 95% CI 0.62–0.96, *P* = 0.02) [15]. Besides, multiple clinical trials exploring other ICI-based combination strategies are ongoing.

## The rationale of PARPi combining ICI therapy

### Tumor mutation burden and neoantigen

The relationship between tumor mutation burden and efficacy of ICI has been confirmed in previous studies [78, 91]. Tumor mutation burden is regarded as a surrogate of neoantigen burden which heralds the therapeutic response after ICI treatment [78]. Tumor mutation burden is closely related with DDR deficiency [92]. Hyper-mutated tumors often harbor one or more mutations in key components of DDR pathways such as hMSH2, BRCA1/2, and POLE [92–94]. After receiving anti-PD-1/PD-L1 treatment, patients with DDR deficiencies had a higher response rate compared with patients without these deficiencies [95]. Thus, patients with HR or other DDR deficiencies might be candidates for both PARPi and ICI therapy.

PARPi-mediated catastrophic DNA damage is a favorable factor for ICI therapy, even though the influence of HR deficiency on tumor mutation burden is weaker than MMR deficiency [92]. After receiving PARPi treatment, accumulated chromosome rearrangements generate plenty of neoantigens and elevate the immunogenicity of tumor. Theoretically, PARPi could increase the sensitivity of patients to ICI therapy by increasing mutation burden.

### DNA damage and cGAS-STING pathway

**Fig. 2:**
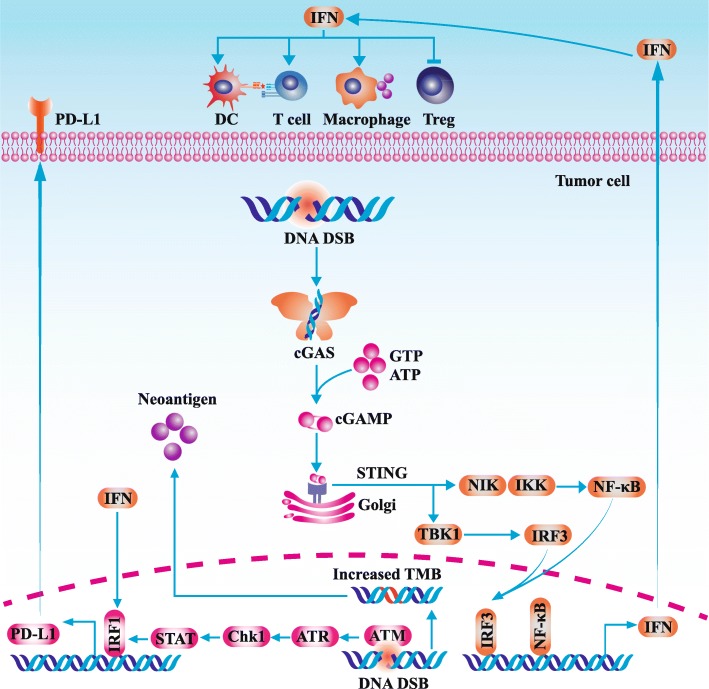
The cross-talk between DNA damage and immune response. Following the stimulation of cytoplasmic dsDNA, cGAS is activated and catalyzes the generation of cyclic-dinucleotide. CDN is a second messenger which promotes the conformational change of STING. Active STING mainly initiates the downstream TBK1-IRF3-Type I IFN pathway. Besides, STING could activate the NF-κB pathway which cooperates with IRF3 to upregulate the generation of type I IFN. Type I IFN has a substantial influence on systemic immune response and regulates multiple components in anti-cancer immunity. Moreover, PARPi treatment-induced double-strand break could upregulate PD-L1 expression by augmented anti-cancer immunity or ATM-ATR-Chk1 pathway. Lastly, after receiving PARPi treatment, accumulated chromosome rearrangements generate plenty of neoantigens and elevate the immunogenicity of tumor. DSB, double-strand break; STING, stimulator of interferon genes; cGAS cyclic GMP-AMP synthase; TMB, tumor mutation burden

Apart from tumor mutation burden, DDR-mediated immune responses collaborate with ICI which remodel tumor immune microenvironment and boost the cancer-immunity cycle [96]. Due to the genomic instability and incomplete DNA repair repertoire, DNA damages accumulate and could not be fully repaired in tumor cells. As a result, these DNA damages persist in a low level which might increase the possibility of the exposure of double-strand DNA (dsDNA) in cytoplasm [97]. Following the stimulation of cytoplasmic dsDNA, cyclic GMP-AMP synthase (cGAS) is activated and catalyzes the generation of cyclic-dinucleotide (CDN) [98]. CDN is a second messenger which promotes the conformational change of stimulator of interferon genes (STING) [99]. Active STING mainly initiates the downstream TBK1-IRF3-Type I IFN pathway [100]. Besides, STING could activate NF-κB pathway which cooperates with IRF3 to upregulate the generation of type I IFN [101]. Type I IFN has a substantial influence on systemic immune response and regulates multiple components in anti-cancer immunity especially DCs, natural killer cells (NKs) and T cells (Fig. 2) [21].

Under the immunostimulatory effect of type I IFN, the cross-presentation capability of DCs and the trafficking and cytotoxicity of T cells are enhanced by various manners [102]. Firstly, by upregulating the synthesis of MHC and co-stimulatory molecules, as well as CXC Chemokine Receptor 7 (CCR7), type I IFN promotes the maturation and lymph node-homing of DCs [103, 104]. Secondly, type I IFN stimulates the secretion of Th1 chemokines such as C-X-C motif chemokine ligand 9 (CXCL9) and C-X-C motif chemokine ligand 10 (CXCL10) which further promote the trafficking of T cells [105]. Thirdly, type I IFN could boost the function of cytotoxic T lymphocytes (CTLs) by increasing the generation of perforin 1 and granzyme B [106]. Moreover, type I IFN stimulates macrophages to secrete various pro-inflammatory cytokines such as interleukin-1β (IL-1β) [107]. In the meanwhile, type I IFN could weaken the immunosuppressive function of Treg by downregulating the level of cyclic AMP (cAMP) [108]. Generally, DNA damage and cytosolic DNA-mediated cGAS-STING pathway facilitate to reshape an immunosupportive environment. A series of studies demonstrated that PARPi-mediated DNA damage could enhance the recruitment and infiltration of T cells into tumor via activating cGAS-STING pathway [109–111]. The preexisting TIL is the prerequisite of tumor-killing effect of ICI, thus PARPi could synergize with ICI and decrease the risk of drug resistance [112].

### PARPi-mediated PD-L1 upregulation

The level of PD-L1 is a vital biomarker predicting the efficacy of anti-PD-1/PD-L1 efficacy [86, 113]. The upregulation of PD-L1 is mainly driven by inflammation; thus, the abundance of PD-L1 could reflect the status of tumor immune microenvironment [114]. In cancer cell lines and mouse models, it was observed that PARPi administration induced PD-L1 upregulation [24]. Further exploration identified that the upregulation of PD-L1 was mainly related to the augmented anti-cancer immunity after PARPi treatment [110]. Besides, Sato et al. reported that X-rays or PARPi-induced double-strand breaks could directly upregulate PD-L1 by ATM-ATR-Chk1 pathway which was independent of IFN pathway [115]. Therefore, additional anti-PD-1/PD-L1 treatment could neutralize the feedback upregulation of PD-L1 and reactivate the blunt tumor-killing activity of TIL.

### PARPi-mediated reprogram of immune microenvironment

The interaction between DDR and immune response is the basis of the combination therapy of PARPi and ICI. In the absence of PARPi, low-level DNA damages at baseline induce the chronic inflammation which promotes the initiation and development of cancers [23]. Actually, the biologic effect of type I IFN is bidirectional which changes along with the timing and magnitude of type I IFN production [116]. Persistent type I IFN secretion at baseline could inhibit the expansion of conventional DCs and upregulates the generation of PD-L1 and IL-10 in DCs and macrophages [116]. PARPi-mediated catastrophic DNA damage and subsequently acute inflammation could rapidly elevate the abundance of type I IFN via cGAS-STING pathway [117]. Contrary to the pro-tumor inflammation at baseline, PARPi-mediated acute inflammation remodels tumor immune microenvironment and drives a systemic Th1-skewing immune response [117]. This transformation boosts the priming of immunity and tumor-killing activity, synergizing with ICI for the renaissance of anti-cancer immunity.

## The preclinical and clinical studies of PARPi combining ICI therapy

Inspired by the synergistic effect of PARPi and ICI treatment, numerous studies are ongoing to explore the actual efficacy of the combination therapy in tumors harboring BRCA1/2 or other DDR genes mutations (Table 1).

**Table 1:** Ongoing clinical trials exploring the efficacy of PARPi combining ICI treatment

Intervention	Clinical Trial	Cancer	Phase	Status
BGB-A317 and BGB-290	NCT02660034	Advanced solid tumors	I	Recruiting
Niraparib and Atezolizumab	NCT03598270	Recurrent ovarian cancer	III	Recruiting
Niraparib and PD-1 inhibitor	NCT03308942	NSCLC	II	Active, not recruiting
Niraparib and Pembrolizumab	NCT02657889	TNBC or ovarian cancer	I/II	Active, not recruiting
Niraparib and TSR-042	NCT03651206	Ovarian cancer and endometrial Cancer	II/III	Not yet recruiting
	NCT03602859	Stage III or IV non-mEOC	III	Recruiting
	NCT03574779	Recurrent ovarian cancer	II	Recruiting
	NCT03307785	Advanced or metastatic solid cancer	I	Recruiting
Olaparib and Atezolizumab	NCT02849496	Advanced or metastatic non-HER2-positive breast cancer	II	Recruiting
Olaparib and Durvalumab	NCT03167619	TNBC	II	Recruiting
	NCT02546661	Muscle invasive bladder cancer	I	Recruiting
	NCT03459846	Stage IV platinum-ineligible Urothelial Cancer	II	Recruiting
	NCT03334617	NSCLC	II	Recruiting
	NCT03851614	MMR proficient colorectal cancer, pancreatic cancer, and leiomyosarcoma	II	Recruiting
	NCT02734004	Advanced ovarian, breast, lung, and gastric cancers	I/II	Recruiting
	NCT02882308	Squamous cell carcinoma of the head and neck	II	Recruiting
	NCT03772561	Advanced solid tumors	I	Recruiting
	NCT02484404	Recurrent ovarian, TNBC, lung, prostate, and colon cancers	I/II	Recruiting
Olaparib and Pembrolizumab	NCT03834519	mCRPC	III	Not yet recruiting
	NCT02861573	mCRPC	I	Recruiting
Olaparib and Tremelimumab	NCT02571725	BRCA deficient Ovarian Cancer	I/II	Recruiting
Olaparib, Durvalumab, and Tremelimumab	NCT02953457	Recurrent or refractory ovarian, fallopian tube or primary peritoneal cancer with BRCA mutation	II	Recruiting
Rucaparib and Atezolizumab	NCT03101280	Advanced gynecologic cancers and TNBC	I	Recruiting
	NCT03694262	Recurrent or progressive endometrial carcinoma.	II	Not yet recruiting
Rucaparib and Nivolumab	NCT03639935	ABC	II	Recruiting
	NCT03572478	Prostate cancer or endometrial cancer	I/II	Recruiting
	NCT03824704	Selected solid tumors*	II	Not yet recruiting
	NCT03522246	Ovarian cancer	III	Recruiting
	NCT03338790	mCRPC	II	Recruiting
	NCT02873962	Relapsed ovarian, fallopian tube or peritoneal cancer	II	Recruiting
SHR-1210 and SHR3162	NCT03182673	Advanced solid tumors	I	Recruiting
Talazoparib and Avelumab	NCT03637491	Advanced or metastatic RAS-mutant solid tumors	II	Recruiting
	NCT03565991	BRCA or ATM mutant tumors	II	Recruiting
	NCT03330405	Advanced or metastatic solid tumors	II	Recruiting

Note: *ABC* advanced or metastatic biliary tract cancer, *mCRPC* metastatic castration-resistant prostate cancer, *mEOC* mucinous epithelial ovarian cancer, *NSCLC* non-small cell lung cancer, *MMR* mismatch repair, *TNBC* triple-negative breast cancer

*Including epithelial ovarian cancer, fallopian tube cancer, primary peritoneal carcinoma, metastatic transitional cell cancer of the renal pelvis and ureter, urothelial carcinoma, high-grade serous carcinoma, endometrioid cdenocarcinoma, etc

### PARPi combining with anti-PD-1/PD-L1 treatment

As early as 2017, Jiao et al. noticed the association between PARP inhibition and treatment-related PD-L1 upregulation [24]. In breast cancer cell lines and xenograft models, PARPi treatment significantly increased the expression of PD-L1 [24]. The results of the co-culture experiment showed that breast cancer cells undergoing Olaparib treatment were resistant to cell-killing activity of activated human peripheral blood mononuclear cells [24]. To further investigate whether additional anti-PD-L1 blockade could overcome PARPi-induced immune suppress in vivo, EMT6 syngeneic mouse models were adopted and received anti-PD-L1 blockade/Olaparib monotherapy or combination therapy [24]. Combination therapy exhibited more potent anti-cancer effect and elevated the abundance of TILs compared with monotherapies [24]. In this study, PARPi-induced PD-L1 upregulation was independent of cGAS-STING-IFN pathway [24].

#### Combination therapy in BRCA1/2 mutated models

Contrary to the observation of Jiao and colleagues, Ding et al. found PARPi treatment activated STING pathway and triggered robust anti-cancer immunity, as well as induced inflammation-mediated PD-L1 upregulation [118]. Researchers designed two genetically engineered mouse models bearing high-grade serous ovarian cancer: PBM (driven by p53 depletion, BRCA1 depletion, and c-Myc overexpression) and PPM (driven by p53 depletion, PTEN depletion, and c-Myc overexpression) [118]. Anti-PD-1 monotherapy showed nonsignificant effect on PBM, while concurrent Olaparib combining with anti-PD-1 treatment significantly retarded tumor growth [118]. Compared with Olaparib monotherapy, mice receiving combination therapy had prolonged survival time [118]. Further exploration in tumor immune microenvironment revealed that the abundance of TIL increased, the expression of negative co-stimulatory molecules (PD-1/Lag-3/Tim-3) decreased, and the secretion of pro-inflammation cytokines (IFN-γ and TNF-α) elevated after Olaparib administration [118]. Besides, the expression of CD80/86 and MHC was upregulated on DCs following Olaparib treatment [118]. In the peripheral blood of mice undergoing Olaparib treatment, CD8^+^ T cells possessed greater capability to produce IFN-γ and TNF-α [118]. PARPi-mediated local and systemic immune response could be abrogated by STING pathway blockade and enhanced by PD-1 inhibitor [118].

#### Combination therapy in BRCA1/2 proficient models

The investigations of combination therapy were mainly conducted in BRCA1/2 mutated tumors [119]. However, it is still controversial that patients without mutations in BRCA or other HR genes could benefit from PARPi combining ICI treatment. Ding et al. found that the combination therapy showed non-significant effect on BRCA-proficient ovarian cancers while Wang et al. found the concurrent ICI treatment remarkably enhanced the efficacy of PARPi in multiple BRCA-proficient tumors [120]. Niraparib combined with anti-PD-1/PD-L1 therapy increased the infiltration of immune cells into tumor bed and slowed the tumor growth in BRCA-proficient breast cancer, sarcoma, lung squamous cell carcinoma, and colon adenocarcinoma, as well as bladder cancer [120]. This combination strategy might conduce to broaden the application of PARPi.

Regardless of BRCA status, Sen et al. interrogated the efficacy of PARPi combining with ICI treatment in small cell lung cancer (SCLC) model [110]. SCLC is a unique cancer which is characterized by TP53 and RB loss, as well as MYC amplification [121]. Dysregulated cell cycle checkpoint leads to increased replication stress [122]. In the meanwhile, the loss of RB in SCLC reduces the transcription inhibition of PARP [92]. The viability of SCLC is highly dependent on hyperactive PARP, thus SCLC is prone to be sensitive to PARPi treatment [92]. By activating the STING pathway, the combination therapy of Olaparib and anti-PD-L1 significantly elevated the abundance of CD3^+^ T cells and CD8^+^ cytotoxic T cells in tumor bed while decreased the infiltration of PD-1^+^/Tim-3^+^ exhausted T cells and CD25^+^/FoxP3^+^ Tregs [110]. Besides, it was detected that chemokines such as CXCL10 and CCL5 increased after the combination therapy [110]. Although neither Olaparib nor anti-PD-L1 monotherapy could retard tumor growth, the combination therapy induced complete tumor regression and sustained a durable anti-cancer effect in all treated mice [110].

#### Combination therapy in ongoing clinical trials

Based on the encouraging results of multiple preclinical studies, a series of clinical trials are ongoing to evaluate the efficacy of PARPi combining ICI treatment in a broad range of cancers. The preliminary data of NCT02484404 showed that the combination therapy of Olaparib and Durvalumab effectively reduced tumor burden (measured by PSA reduction > 50%) in 8/17 unselected metastatic castrate-resistant prostate cancer [123]. Mutation in DDR was a favorable biomarker indicating better response to the combination therapy (12-month progression-free survival probability of deficient DDR vs. proficient DDR, 83.3% vs. 36.4%, *P* = 0.03) [123, 124]. Besides, the results of SCLC cohort of NCT02484404 indicated the baseline TIL status also affected the efficacy of combination strategy [125].

Another clinical study (phase II NCT02734004) explored the effect of Olaparib and Durvalumab combination scheme in germline BRCA-mutated platinum-sensitive relapsed ovarian cancer patients [126]. The interim results indicated this combination strategy was well tolerated. In the meanwhile, the disease control rate (DCR) at 12 weeks was 81% and the objective response rate (ORR) was 63% [126]. These early data strongly support the feasibility of the combination scheme containing Olaparib and Durvalumab.

Apart from Olaparib plus Durvalumab strategy, a phase II study NCT02657889 evaluated the effect of Niraparib combining with Pembrolizumab therapy in metastatic triple-negative breast cancer and recurrent ovarian cancer patients [127, 128]. Compared with the therapeutic response in overall enrolled patients, ORR was markedly higher in BRCA1/2 mutated patients (ORR of BRCA1/2 mutated vs. overall patients in breast cancer cohort, 67% vs. 29%; ORR of BRCA1/2 mutated vs. overall patients in ovarian cancer cohort, 45% vs. 25%) [127, 128].

### PARPi combining with anti-CTLA-4 treatment

Compared with the intensive attention to anti-PD-1/PD-L1, combination scheme of PARPi and anti-CTLA-4 was rarely studied. In 2015, Higuchi et al. conducted a preclinical study to explore the efficacy of PARPi combining with anti-CTLA-4 treatment in BRCA1 deficient ovarian cancer model [129]. In vitro experiment, researchers found that PARPi-induced apoptosis increased when tumor cells were exposed to additional IFN-γ or TNF-α [129]. In vivo experiment, anti-CTLA-4 combining with PARPi treatment significantly increased the proportion of effector/memory CD8^+^ T cells in tumor microenvironment [129]. Further investigation showed this combination therapy remarkably upregulated the generation of cytokines in TILs [129]. On the contrary, neither anti-CTLA-4 nor PARPi monotherapy could significantly change the abundance and function of lymphocytes [129]. Compared with monotherapy, the combination treatment completely eliminated visible tumor mass and maintained a long-term tumor-free survival in most of mice [129]. The anti-tumor effect of combination therapy was impaired by anti-IFN-γ neutralizing antibody [129]. Subsequently adoptive immune cell transfer experiment confirmed the synergistic effect of combination therapy was highly dependent on T cell-mediated immune response [129]. Recipient mice receiving CD8^+^ splenocytes from donor mice undergoing combination treatment exhibited the resistance to the following tumor challenge and survived longer than the untreated control group [129]. Combination therapy-mediated protective immune memory contributed to a durable anti-tumor effect [129].

## Conclusion

DDR deficiency is the driving factor and an essential component of carcinogenesis. As the Achilles’ heel of cancer cells, DDR deficiency is an ideal treatment target to interfere genome stability and induce tumor cell death. Based on the synthetic lethal effect, PARPi was initially designed for BRCA deficient patients. Then, it was revealed that PARP inhibition and entrapment induced cytosolic dsDNA formation and subsequent cGAS-STING pathway activation. The cross-talk between PARPi and immune response is the fundament of the combination therapy of PARPi and ICI. Preclinical results and early data of ongoing clinical trials indicated the synergistic effect of PARPi and ICI treatment. By the combination scheme with concurrent ICI, the application of PARPi might be extended to a broad range of cancers far beyond BRCA deficient phenotype. In the meanwhile, PARPi could substantially modulate anti-cancer immune response, enhance immune priming, and reinforce the tumor-killing activity. We believe PARPi is a potential sensitizer for ICI treatment and this novel combination is meaningful for cancer immunotherapy in the future.

## Data Availability

Data sharing not applicable to this article as no datasets were generated or analyzed during the current study.

## Abbreviations

BER: Base excision repair
cAMP: Cyclic AMP
CCR7: CXC Chemokine Receptor 7
CDN: Cyclic-dinucleotide
cGAS: Cyclic GMP-AMP synthase
CTL: Cytotoxic T lymphocyte
CTLA-4: Cytotoxic T-lymphocyte-associated protein 4
CXCL10: C-X-C motif chemokine ligand 10
CXCL9: C-X-C motif chemokine ligand 9
DC: Dendritic cell
DCR: Disease control rate
DDR: DNA damage response
DSB: Double-strand break repair
dsDNA: Double-strand DNA
HR: Homologous recombination
ICI: Immune checkpoint inhibitor
IL-1β: Interleukin-1β
ITIM: Immunoreceptor tyrosine-based inhibitory motif
ITSM: Immunoreceptor tyrosine-based switch motif
MHC: Major histocompatibility complex
MMR: Mismatch repair
NER: Nucleotide excision repair
NHEJ: Nonhomologous end joining
NK: Natural killer
ORR: Objective response rate
PARP: Poly ADP-ribose polymerase
PARPi: PARP inhibitor
PD-1: Programmed cell death-1
pMHC: Peptide-major histocompatibility complex
SCLC: Small cell lung cancer
SSB: Single-strand break
STING: Stimulator of interferon genes
TAA: Tumor-associated antigens
TCR: T cell receptor
TIL: Tumor-infiltrating lymphocyte
Treg: Regulatory T cell
